# Morbidity and mortality outcomes of COVID-19 patients with and without hypertension in Lagos, Nigeria: a retrospective cohort study

**DOI:** 10.1186/s41256-021-00210-6

**Published:** 2021-07-29

**Authors:** Akin Abayomi, Akin Osibogun, Oluchi Kanma-Okafor, Jide Idris, Abimbola Bowale, Ololade Wright, Bisola Adebayo, Mobolanle Balogun, Segun Ogboye, Remi Adeseun, Ismael Abdus-Salam, Bamidele Mutiu, Babatunde Saka, Dayo Lajide, Sam Yenyi, Rotimi Agbolagorite, Oluwatosin Onasanya, Eniola Erinosho, Joshua Obasanya, Olu Adejumo, Sunday Adesola, Yewande Oshodi, Iorhen E Akase, Shina Ogunbiyi, Adenike Omosun, Femi Erinoso, Hussein Abdur-Razzaq, Nike Osa, Kingsley Akinroye

**Affiliations:** 1 Lagos State Ministry of Health/Lagos Incident Management Command System, Lagos, Nigeria; 2 College of Medicine University of Lagos, Idi-Araba, Lagos, Nigeria; 3 Lagos State Primary Health Care Board, Lagos, Nigeria; 4 Mainland Hospital, Yaba, Lagos, Nigeria; 5 Lagos State University College of Medicine, Lagos, Nigeria; 6 Lagos State Biobank, Lagos, Nigeria; 7 World Health Organization, Nigeria Office, Abuja, Nigeria; 8 Nigeria Centre for Disease Control, Abuja, Nigeria; 9 Lagos University Teaching Hospital, Lagos, Nigeria; 10 Lagos State University Teaching Hospital, Lagos, Nigeria; 11 Nigerian Heart Foundation, Lagos, Nigeria

**Keywords:** COVID-19, Nigeria, Hypertension, Comorbidities, Coronavirus, SARS-CoV-2 virus, Pandemic

## Abstract

**Background:**

The current pandemic of coronavirus disease (COVID-19) caused by the severe acute respiratory syndrome coronavirus 2 (SARS-CoV-2) has shown epidemiological and clinical characteristics that appear worsened in hypertensive patients. The morbidity and mortality of the disease among hypertensive patients in Africa have yet to be well described.

**Methods:**

In this retrospective cohort study all confirmed COVID-19 adult patients (≥18 years of age) in Lagos between February 27 to July 62,020 were included. Demographic, clinical and outcome data were extracted from electronic medical records of patients admitted at the COVID-19 isolation centers in Lagos. Outcomes included dying, being discharged after recovery or being evacuated/transferred.

Descriptive statistics considered proportions, means and medians. The Chi-square and Fisher’s exact tests were used in determining associations between variables. Kaplan–Meier survival analysis and Cox regression were performed to quantify the risk of worse outcomes among hypertensives with COVID-19 and adjust for confounders. *P*-value ≤0.05 was considered statistically significant.

**Results:**

A total of 2075 adults with COVID-19 were included in this study. The prevalence of hypertension, the most common comorbidity, was 17.8% followed by diabetes (7.2%) and asthma (2.0%). Overall mortality was 4.2% while mortality among the hypertensives was 13.7%. Severe symptoms and mortality were significantly higher among the hypertensives and survival rates were significantly lowered by the presence of additional comorbidity to 50% from 91% for those with hypertension alone and from 98% for all other patients (*P* < 0.001). After adjustment for confounders (age and sex), severe COVID-19and death were higher for hypertensives {severe/critical illness: HR = 2.41, *P* = 0.001, 95%CI = 1.4–4.0, death: HR = 2.30, *P* = 0.001, 95%CI = 1.2–4.6, for those with hypertension only} {severe/critical illness: HR = 3.76, *P* = 0.001, 95%CI = 2.1–6.4, death: crude HR = 6.63, *P* = 0.001, 95%CI = 3.4–1.6, for those with additional comorbidities}. Hypertension posed an increased risk of severe morbidity (approx. 4-fold) and death (approx. 7-fold) from COVID-19 in the presence of multiple comorbidities.

**Conclusion:**

The potential morbidity and mortality risks of hypertension especially with other comorbidities in COVID-19 could help direct efforts towards prevention and prognostication. This provides the rationale for improving preventive caution for people with hypertension and other comorbidities and prioritizing them for future antiviral interventions.

**Supplementary Information:**

The online version contains supplementary material available at 10.1186/s41256-021-00210-6.

## Introduction

Globally, there is an ongoing pandemic of Coronavirus disease (COVID-19), an infectious disease caused by a newly discovered coronavirus called the Severe Acute Respiratory Syndrome Coronavirus 2 (SARS-CoV-2) [1]. The infection fatality rate (IFR) of this disease has been estimated at a range of around 0.5–1% [2, 3], with higher rates among those aged 60 or older [4]. The majority of cases of COVID-19 experience mild to moderate respiratory illness and recover with supportive care. Serious illness is more likely with the elderly and those with underlying comorbidities like cardiovascular disease, chronic respiratory disease, diabetes, and cancer [5, 6].

Much earlier in a study in China, where the COVID-19 was first reported, it was found that 48% of patients had a comorbidity, with hypertension being the most prevalent (30%), followed by diabetes (19%) and coronary heart disease (8%) [7]. In a study from Italy, it was reported that COVID-19 deaths were mostly among people with comorbidities (99%), the majority of these were hypertensive (76.5%) [8, 9]. Studies have shown that hypertension imposes on those who suffer from it an increased risk of getting infected with COVID-19, experiencing worse symptomatology and complications and a 2-fold risk of dying from the infection. Hypertension was reported to have had a hazard ratio (HR) of 1.70 [95% confidence interval (CI) 0.92–3.14] to 3.05 (95% CI 1.57–5.92) for mortality in some unadjusted epidemiological studies in China [7, 10].

High blood pressure is common among people over 60 years of age, the prevalence being nearly as high as two-thirds of this population. Long-term ill health and aging lead to a weakened immune system increasing the susceptibility of people with chronic illnesses to coronavirus infection. Along with the increased risk of infection and worsened outcomes among hypertensives, there is a growing concern that some medications used in the treatment may influence mortality in patients with COVID-19 [11, 12]. These medications such as angiotensin-converting enzyme (ACE) inhibitors and angiotensin receptor blockers (ARBs), cause a rise in blood levels of ACE2 [13]. The theory is that the COVID-19 virus infects human cells by forming a bond with ACE2, a requirement for viral entry into host cells [ 14], thus increasing individual susceptibility to infection and propagation of the virus [15]. Several other studies have however found no association between the use of these drugs and the severity of COVID-19 [16].

The epidemiological and clinical characteristics of patients with COVID-19 in terms of the detailed clinical course of illness, risk factors for mortality its spread and even its treatment are still being studied and documented. Understanding the potential effect of hypertension on the risk of mortality from COVID-19 could help clinicians to identify and characterize patients’ prognoses at an early stage so as to provide timely intervention. This study, hence, was aimed at assessing the hypothesis that hypertension worsens the morbidity and mortality outcomes of confirmed COVID-19 patients.

## Methods

### Study subjects and design

This retrospective observational study was conducted using data collected from 2075 adult COVID-19 patients (≥18 years of age) consecutively admitted across ten designated isolation and treatment centers and hospitals, with reverse transcription polymerase chain reaction (RT-PCR) test results confirming COVID-19. These patients received care at hospitals or isolation and treatment centers dedicated solely to the treatment of COVID-19, in Lagos, Nigeria from 27 February to 6 July 2020. This study was conducted to include only patients who had been admitted to care at the isolation centers as at the commencement of the study on 6 July 2020. The strategy at that phase of the outbreak was containment, in the attempt to reduce community spread. Therefore, all identified positive cases were admitted to the isolation centers.

### Data collection

Patients’ data collected on admission included sociodemographic data, details of their medical history and comorbidities, symptoms, severity of symptoms on admission, clinical outcomes and status at the end of the study (recovery, transfer/evacuation or death). Data was collected at the hospital/isolation center using the electronic medical records created specifically for the Lagos State COVID-19 response. Data extracted for the purpose of this study was completely anonymized.

### Description of variables

Sociodemographic data included the age, sex, health facility and epidemiological identifier. Details of their medical history were limited to the reported comorbidities. The patients’ presenting symptoms were recorded and the severity of symptoms on admission was categorized as mild, moderate, severe, or critical. Asymptomatic patients were categorized as mild, while cases with cough, fever, respiratory rate < 30 breaths per minute and peripheral capillary oxygen saturation (spO2) > 90% were categorized as moderate. Patients who had grunting respiration, respiratory rate > 30 breaths per minute and spO2 < 90% on admission were classified as severe. The patients categorized as critical cases were those in respiratory failure [17].

### History of hypertension

The data on comorbidities including hypertension was based on the patient’s report of the previous diagnosis prior to the infection with SARS-CoV-2. Those who required antihypertensive medication during hospitalization with no prior prescription were treated with antihypertensives, while those who had been on medications prior to admission, were treated with their usual prescribed medication. These patients were not stratified according to whether or not they were receiving antihypertensive while on admission.

### Outcomes

The major outcomes after admission were discharged following recovery or death. Patients still receiving care as of July 6, 2020, who had neither been discharged, transferred nor had died were classified as ‘yet undetermined’. Details of follow-up of patients after leaving the hospital or isolation center were not included in this dataset.

### Data management and analysis

Data were analyzed using the SPSS version 20 and presented in frequencies and proportions. Descriptive statistics considered means ± standard deviation (SD) for continuous variables that were normally distributed and median ± interquartile range (IQR) for those identified as skewed. Bivariate analyses (Chi-square test (trend and non-trend) and the Fisher’s exact test as required) were used in determining associations between variables. Kaplan–Meier survival analysis was done to compare mortality between hypertensives and patients without hypertension. The Cox proportional hazards model was used to quantify the risk of worse outcomes among hypertensives with COVID-19 and adjust for the effect of confounders. *p*-value < 0.05 was considered statistically significant.

## Results

The patients were predominantly less than 40 years of age and about a tenth of them were over 60 years of age. The median age of the patients was 40 (IQR = 32–50) years, and the oldest was 98 years of age. The male to female ratio was 2:1. About a quarter (23.3%) of the patients had at least one comorbidity including hypertension, other cardiovascular (CVS) diseases, diabetes, asthma, HIV, Hepatitis B, cancer, renal disease, sickle cell disease, tuberculosis and other lung diseases, while 17.8% had hypertension alone. Over 50% of them were asymptomatic or mildly symptomatic at the time of admission. Severity on admission ranged between mild to critical and over half of them (56.9%) had mild symptoms; about 2% of them were in critical condition on admission and at the end of the study period, there was about 4% mortality (Table 1).

**Table 1:** Patient characteristics

Variable	Frequency (***N*** = 2075)	%
**Age** (in years)		
< 40	1017	49.0
40–49	526	25.3
50–59	321	15.5
> −60	211	10.2
**Median age (IQR), min-max**	40 (32–50), 18–98	
**Sex**		
Male	1379	66.5
Female	696	33.5
**Comorbidities (*n*** **= 2071**^**a**^**)**		
Yes	483	23.3
No	1588	76.7
**Type of comorbidity**^**d**^		
Hypertension	369	17.8
Diabetes	150	7.2
Asthma	42	2.0
HIV/Hepatitis B	15	0.7
Other CVS diseases	14	0.6
Cancer	15	0.7
Renal disease	10	0.5
Sickle cell disease	6	0.3
Tuberculosis & other lung diseases	7	0.3
**Symptoms (*n*** **= 2071**^**b**^**)**		
Asymptomatic to mild	1192	57.6
Symptomatic beyond mild	879	42.4
**Severity on admission (*n*** **= 2071**^**c**^**)**		
Mild/asymptomatic	1179	56.9
Moderate	743	35.9
Severe	107	5.2
Critical	42	2.0
**Discharge status**		
Determined	1739	83.8
Yet undetermined (still on admission)	336	16.2
**Discharge status determined (*n*** **= 1739)**		
Died	73	4.2
Recovered	1638	98.4
Transferred out/Evacuated	28	1.6

^a^ Missing = 4(0.2%) ^b^Missing = 4(0.2%) ^c^Missing = 4 (0.2%)

^d^ Multiple comorbidities reported by some patients

When the groups were stratified by the presence or absence of hypertension it was found that hypertensive cohorts were significantly older (55.68 ± 12.9 vs 38.68 ± 11.5) and there was an increasing proportion of hypertensives across the age groups (p for trend = 0.001). Both cohorts were proportionately similar in sex distribution. A significant proportion of the hypertensive cohort suffered the worse forms of COVID-19; severe (14.4% vs 3.2%) and critical (6.8% vs 1.0%), compared to the no-hypertension cohort (p for trend < 0.001). The time till the endpoint of admission irrespective of outcome was significantly different between both cohorts. The hypertensive group experienced a relatively shorter time on admission before the final outcome, with a median duration of admission shorter for hypertensives than for patients without hypertension (12(IQR = 8–14), 13(IQR = 10–14), respectively). The disease outcome was significantly different between the cohorts; 13.7% of those who were hypertensive died compared to 2.2% of patients without hypertension (*p* = 0.001) (Table 2).

**Table 2:** Comparison of patients’ characteristics and morbidity/ mortality against hypertensive status

Variable	Hypertensive(n(%))			X^**2**^	***P***-value
	Yes	No	Total		
**Age** (in years)				457.47^c^	0.001
< 40	33 (8.9)	983 (57.8)	1016 **(49.1)**		
40–49	96 (26.0)	429 (25.2)	525 **(25.4)**)		
50–59	118 (32.0)	203 (11.9)	321 **(15.5)**		
> −60	122 (33.1)	87 (5.1)	209 **(10.1)**		
**Total**	**369 (100.0)**	**1702 (100.0)**	**2071 (100.0)**		
**Median age (IQR)**	55 (47–63)	37 (30–45)	40 (32–50)		
**Mean ± SD**	**55.62 ± 12.9**	**38.68 ± 11.5**	**41.72 ± 13.4**	t = 23.3	0.001
**Sex**				0.47	0.492
Male	251 (68.0)	1126 (66.2)	1377 (64.5)		
Female	118 (32.0)	576 (33.8)	694 (33.5)		
**Total**	**369 (100.0)**	**1702 (100.0)**	**2071 (100.0)**		
**Severity on admission**				159.87^c^	< 0.001
Mild	136 (36.9)	1043 (61.3)	1179 (56.9)		
Moderate	155 (42.0)	588 (34.5)	743 (35.9)		
Severe	53 (14.4)	54 (3.2)	107 (5.2)		
Critical	25 (6.8)	17 (1.0)	42 (2.0)		
**Total**	**369 (100.0)**	**1702 (100.0)**	**2071 (100.0)**		
**Time till endpoint**				5.24^c^	0.022
< 14 days	197 (66.1)	860 (59.8)	1057 (60.9)		
14–28	100 (33.6)	557 (38.7)	657 (37.8)		
> 28 days	1 (0.3)	22 (1.5)	23 (1.3)		
**Total**	**298 (100.0)**	**1439 (100.0)**	**1737**^**a**^**(100.0)**		
**Median time (IQR)**	12 (8–14)	13 (10–14)			
**Mean ± SD**	11.06 ± 5.47	12.4 ± 4.91			
**Discharge status determined**				95.70	< 0.001
Died	41 (13.7)	32 (2.2)	73 (4.2)		
Recovered	247 (82.3)	1391 (96.7)	1638 (94.2)		
Transferred out/evacuated	12 (4.0)	16 (1.1)	28 (1.6)		
**Total**	**300 (100.0)**	**1439 (100.0)**	**1739**^**b**^**(100.0)**		

^a^Missing = 334(16.1%) ^**b**^

^c^Chi-square test for trend

There was a statistically significant difference in mortality and survival among hypertensive patients who had hypertension only and those hypertensives with multiple comorbidities. Similarly, the time till the endpoint was significantly different between those who died and those who survived. A higher proportion of hypertensives that had at least one other comorbidity died (27.5%) compared to those who had hypertension alone (8.1%) (*P* < 0.001). The proportion of hypertensives who died within the first 14 days (21.0%) was higher compared to those whose deaths occurred beyond 2 weeks (2.0%), implying that that death among hypertensives occurred mostly within the first 2 weeks of admission (*P* < 0.001). A greater proportion (26.4%) of the hypertensives with other comorbidities died within the first weeks of admission compared to those who had hypertension only (16.2%) (*P* = 0.086), (Table 3).

**Table 3:** Mortality among COVID-19 hypertensive patients (with or without other comorbidities, < or > 2 week of admission)

Variable	Died(n(%))	Survived (Recovered) (n(%))	Total(n(%))	Χ^**2**^	***P***
**Number of comorbidities**				19.09	< 0.001
1 (Hypertension only)	16 (8.1)	181 (91.9)	197 (100.0)		
> 2	25 (27.5)	66 (72.5)	91 (100.0)		
**Total**	**41 (14.2)**	**247 (85.8)**	**288 (100.0)**		
**Time till endpoint**					< 0.001*
< 14 days	39 (21.0)	147 (79.0)	186 (100.0)		
≥ 14–28	2 (2.0)	100 (98.0)	101 (100.0)		
**Total**	**41 (14.2)**	**247 (85.8)**	**288 (100.0)**		
**Endpoint within 2 weeks (*n*** **= 186)**					
Hypertension only	16 (16.2)	83 (83.8)	99 (100.0)	2.95	0.086
Hypertension with other comorbidities	23 (26.4)	64 (73.6)	87 (100.0)		
**Total**	**39 (21.0)**	**147 (79.0)**	**186 (100.0)**		

*Fishers exact p

**Fig. 1:**
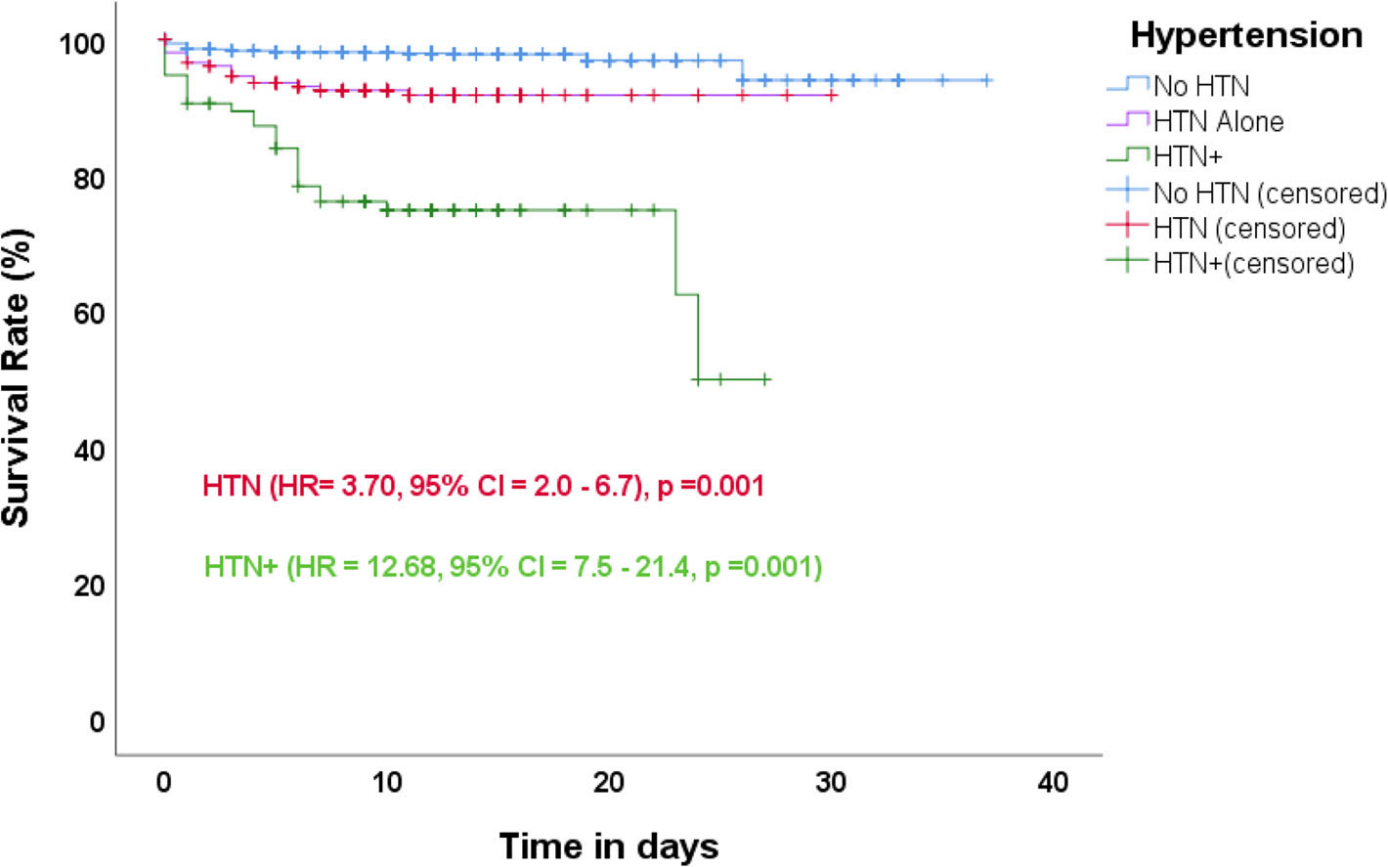
Kaplan–Meier survival curves for mortality among COVID-19 patients with and without hypertension or with additional comorbidities

The Kaplan–Meier estimates indicated that the COVID-19 survival rate for the patients without hypertension was 94, 91% for patients with hypertension only and 50% for those with hypertension with other comorbidities. The log-rank test indicated that there was a statistically significant difference between the three survival rates (*p* < 0.001). The unadjusted hazard ratio (HR) indicated that in the risk of death there was a 4-fold increase among hypertensives and a 13-fold increase among hypertensives with additional comorbidities compared to those who were not hypertensive. Collectively, these results suggest that patients in the hypertensive group were less likely to survive (Fig. 1).

In both the unadjusted and adjusted multivariate analysis (adjusting for sex and age), Cox regression showed that the hypertensive groups had increased rates of severe COVID-19 and mortality. Prior to adjustment, severe/critical illness and death from COVID-19 were significantly associated with being hypertensive (severe/critical illness: crude HR = 4.21, *p* = 0.001, 95%CI = 2.7–6.5) (death: crude HR = 3.70, *p* = 0.001, 95%CI = 2.0–6.7) and having an additional comorbidity (severe/critical illness: crude HR = 7.35, *p* = 0.001, 95%CI = 4.5–11.8) (death: crude HR = 12.68, *p* = 0.001, 95%CI = 7.5–21.4). After adjustment for confounders, the HR for severe illness and death were still higher than for patients without hypertension (severe/critical illness: aHR = 2.41, *p* = 0.001, 95%CI = 1.4–4.0, death: aHR = 2.30, *p* = 0.001, 95%CI = 1.2–4.6, for those with hypertension only) (severe/critical illness: aHR = 3.76, *p* = 0.001, 95%CI = 2.1–6.4, death: aHR = 6.63, *p* = 0.001, 95%CI = 3.4–1.6, for those with additional comorbidities). The hypertension-only patients were about 2 times as likely as patients without hypertension to develop severe disease and 2 times as likely as patients without hypertension to die while those with additional comorbidities were about 4 times as likely as those without hypertension to develop severe disease and about 7 times as likely to die of COVID-19 compared to those without hypertension (Table 4).

**Table 4:** Cox regression for risk of increased severity and death among patients with hypertension compared with patients without hypertension. (adjusting for sex and age)

Variable		Unadjusted			Adjusted	
	HR	95%CI	***p***-value	aHR	95%CI	***p***-value
Severity (Severe/critical)						
HTN	4.21	2.7–6.5	**0.001**	2.41	1.4–4.0	**0.001**
HTN+	7.35	4.5–11.8	**0.001**	3.76	2.1–6.4	**0.001**
Outcome (death)						
HTN	3.70	2.0–6.7	**0.001**	2.30	1.2–4.6	**0.019**
HTN+	12.68	7.5–21.4	**0.001**	6.63	3.4–12.6	**0.001**

*HTN* hypertension, *HTN+* hypertension plus other comorbidities, *HR* Hazard ratio, *aHR* Adjusted hazard ratio

## Discussion

The coronavirus disease (COVID-19) is relatively new and hence understudied. However, the available data has identified the importance of hypertension in the morbidity and mortality picture of the disease. The age and sex distribution found in this study are similar to the findings of a meta-analysis of the clinical characteristics and comorbidities among 1786 coronavirus patients with a median age of 41 years and a male to female ratio of 1.4:1 [18]. The same study found a hypertension prevalence of 15.8%, lower than was found in this study (17.8%). Another study in Wuhan, China found almost a 2-fold higher prevalence [19]. However, in all three studies, the spectrum of comorbidities was the same and hypertension was the most common comorbidity.

Reports of increased incidence and severity of COVID-19 have stated that the severity is skewed towards the elderly population who have a higher prevalence of hypertension and are apparently at particular risk of being infected with the SARS-CoV-2 virus [20]. This study found that severity is related to hypertension and that the hypertensive group also experienced a significantly shorter time on admission before the final outcome. This could be explained by the significantly higher proportion of worse disease outcomes (death) among the hypertensives. While there is an overrepresentation of hypertension among hospitalized and critically ill COVID-19 patients, expert reports have expressed uncertainty whether hypertension is more causal or if other confounders such as age and other comorbidities associated with hypertension augment its role [21]. In the current study, adjustment for confounders meant that patients with hypertension were at a greater risk of increased severity and death from COVID-19 as was seen in a much smaller study in Wuhan, China [22]. Meanwhile, even though it was found in another study that there was a significant two-fold higher risk of mortality due to hypertension when compared with patients with no hypertension [16], the current study found in addition, a significant difference in the potential of dying or surviving among hypertensives to be augmented in the presence of at least one additional comorbidity.

To corroborate the finding of this study that hypertension posed a greater risk of death among COVID-19 patients, a study in China reported that chronic hypertension was more frequent among COVID-19 patients who died compared with those who recovered [23]. Also similar to the finding that hypertension had an HR of 3.70 (crude) for death in 369 patients admitted for COVID-19 another study found an HR of 3.05 in 191 hypertensive patients with COVID-19 [19]. Another study, however, found that hypertension has a lower HR of 1.70 for death in 201 patients with COVID-19 [12]. The implications of the findings of this study are that hypertension is disproportionately more frequently found among COVID-19 patients than other comorbidities and that severe COVID-19 disease and a relatively shorter time to death are associated with being hypertensive, especially in patients who have other comorbidities.

This study was limited because data on hypertensive medication were not included in the dataset. It would have been useful to consider this because there is currently limited clinical evidence of the influence of antihypertensive medication on the prognosis of COVID-19. Nevertheless, continuing a patient’s usual antihypertensive treatment is recommended [24]. Also, the patients in this study were only studied till the end of their stay in the COVID-19 isolation ward. It would have been interesting and beneficial to study the patient beyond the time of discharge.

## Conclusion

The role of other comorbidities worsening the severity and outcomes of COVID-19 has been highlighted in this study. This recognition of the potential morbidity and mortality risks of hypertension especially with other comorbidities in COVID-19 could help direct efforts towards prevention and prognostication. This provides the rationale for improving preventive caution for people with hypertension and other comorbidities and prioritizing them for future antiviral interventions. Studies that demonstrate causation would be beneficial as understanding about COVID-19 improvements. Until more information is available to guide the treatment and management of COVID-19 patients with hypertension, it is important to control blood pressure according to current clinical practice guidelines.

## Supplementary Information

**Additional file 1: Table S1.** Cox regression for risk of death among patients with hypertension compared with patients without hypertension**.** (adjusting for Age, sex, diabetes mellitus, renal diseases, HIV/HBV co-infection, asthma, other cardiovascular diseases and cancer). **Table S2.** Distribution of comorbidities in patients with and without hypertension.

## Data Availability

The datasets generated and/or analyzed during the current study are not publicly available because of ethical restrictions but are available from the corresponding author on reasonable request.

## Abbreviations

ACE: Angiotensin-converting enzyme
ARBs: Angiotensin receptor blockers
HTN: Hypertension
HTN +: Hypertension with other comorbidities
HR: Hazard ratio
aHR: Adjusted hazard ratio

## References

[1] Wu Z, McGoogan JM. Characteristics of and important lessons from the coronavirus disease 2019 (COVID-19) outbreak in China: summary of a report of 72314 cases from the Chinese Center for Disease Control and Prevention. JAMA. 2020;323(13):1239–42. 10.1001/jama.2020.2648 32091533

[2] Mallapaty S. How deadly is the coronavirus? Scientists are close to an answer. Nature. 2020;582:467–8. 10.1038/d41586-020-01738-2 32546810

[3] Russell TW, Hellewell J, Jarvis CI, van Zandvoort K, Abbott S, Ratnayake R, et al. Estimating the infection and case fatality ratio for coronavirus disease (COVID-19) using age-adjusted data from the outbreak on the Diamond Princess cruise ship, February 2020. Euro Surveill. 2020;25(12):2000256. 10.2807/1560-7917.ES.2020.25.12.2000256 PMC7118348 32234121

[4] Verity R, Okell LC, Dorigatti I, Winskill P, Whittaker C, Imai N, et al. Estimates of the severity of coronavirus disease 2019: a model-based analysis. Lancet Infect Dis. 2020;20(6):669–77. 10.1016/S1473-3099(20)30243-7 PMC7158570 32240634

[5] World Health Organization. Coronavirus. Available at: https://www.who.int/health-topics/coronavirus#tab=tab_1. Accessed 6 Aug 2020.

[6] Fang L, Karakiulakis G, Roth M (2020). Are patients with hypertension and diabetes mellitus at increased risk for COVID-19 infection? [published correction appears in Lancet Respir Med. 2020 Jun;8(6):e54]. Lancet Respir Med.

[7] Zhou F, Yu T, Ronghui D, Fan G, Liu Y, Liu Z (2020). Clinical course and risk factors for mortality of adult inpatients with COVID-19 in Wuhan, China: a retrospective cohort study. Lancet.

[8] Istituto Superiore di Sanità. Report sulle caratteristiche dei pazienti deceduti positivi a COVID-19 in Italia Il presente report è basato sui dati aggiornati al 13 Marzo 2020. https://www.epicentro.iss.it/coronavirus/bollettino/Report-COVID-2019_17_marzo-v2.pdf. Accessed 6 Aug 2020.

[9] Doorn-Khosrovani SBW, Roy AP. Rapid Response: COVID-19: learning from the claim data of those who were severely affected but also from their asymptomatic household members. Available at: https://www.bmj.com/content/368/bmj.m1086/rr-1. Accessed 6 Aug 2020.

[10] Wu C, Chen X, Cai Y, Xia J, Zhou X, Xu S, et al. Risk factors associated with acute respiratory distress syndrome and death in patients with coronavirus disease 2019 pneumonia in Wuhan, China. JAMA Intern Med. 2020. 10.1001/jamainternmed.2020.0994. 10.1001/jamainternmed.2020.0994 PMC7070509 32167524

[11] Vaduganathan M, Vardeny O, Michel T, McMurray JJV, Pfeffer MA, Solomon SD (2020). Renin–angiotensin–aldosterone system inhibitors in patients with Covid-19. N Engl J Med.

[12] Patel AB, Verma A (2020). COVID-19 and angiotensin-converting enzyme inhibitors and angiotensin receptor blockers: what is the evidence?. JAMA.

[13] Vuille-dit-Bille RN, Camargo SM, Emmenegger L, Sasse T, Kummer E, Jando J (2015). Human intestine luminal ACE2 and amino acid transporter expression increased by ACE-inhibitors. Amino Acids.

[14] Hoffmann M, Kleine-Weber H, Schroeder S, Kru¨ger N, Herrler T, Erichsen S, et al. (2020). SARS-CoV-2 cell entry depends on ACE2 and TMPRSS2 and is blocked by a clinically proven protease inhibitor. Cell.

[15] Wrapp D, Wang N, Corbett KS, Goldsmith JA, Hsieh CL, Abiona O, Graham BS, McLellan JS (2020). Cryo-EM structure of the 2019-nCoV spike in the prefusion conformation. Science.

[16] Gao C, Cai Y, Zhang K, Zhou L, Zhang Y, Zhang X, Li Q, Li W, Yang S, Zhao X, Zhao Y, Wang H, Liu Y, Yin Z, Zhang R, Wang R, Yang M, Hui C, Wijns W, McEvoy JW, Soliman O, Onuma Y, Serruys PW, Tao L, Li F (2020). Association of hypertension and antihypertensive treatment with COVID-19 mortality: a retrospective observational study. Eur Heart J.

[17] Bowale A, Abayomi A, Idris J, Omilabu S, Abdus-Salam I, Adebayo B (2020). Clinical presentation, case management and outcomes for the first 32 COVID-19 patients in Nigeria. Pan Afr Med J.

[18] Paudel SS. A meta-analysis of 2019 novel coronavirus patient clinical characteristics and comorbidities. Res Square. 2020. 10.21203/rs.3.rs-21831/v1 Accessed 18 Apr 2020, https://www.researchsquare.com/article/rs-21831/v1.

[19] Zhou F, Yu T, Du R, et al. Clinical course and risk factors for mortality of adult inpatients with COVID-19 in Wuhan, China: a retrospective cohort study. Lancet. 2020;395(10229):1054–62. 10.1016/S0140-6736(20)30566-3 PMC7270627 32171076

[20] Schiffrin EL, Flack JM, Ito S, Muntner P, Webb RC (2020). Hypertension and COVID-19. Am J Hypertens.

[21] Arjun Kanwal A, Agarwala A, Martin LW, Handberg EM, Yang E. COVID-19 and hypertension: what we know and don't know. Am Coll Cardiol Expert Anal. https://www.acc.org/latest-in-cardiology/articles/2020/07/06/08/15/covid-19-and-hypertension. Accessed 7 Aug 2020.

[22] Shi S, Qin M, Shen B, Cai Y, Liu T, Yang F, et al. Association of cardiac injury with mortality in hospitalized patients with COVID-19 in Wuhan, China. JAMA Cardiol. 2020. 10.1001/jamacardio.2020.0950. 10.1001/jamacardio.2020.0950 PMC7097841 32211816

[23] Chen T, Wu D, Chen H, Yan W, Yang D, Chen G, et al. Clinical characteristics of 113 deceased patients with coronavirus disease 2019: retrospective study. BMJ 2020;368:m1091. Impact of antihypertensive treatments in COVID-19 2065. Downloaded from https://academic.oup.com/eurheartj/article-abstract/41/22/2058/5851436 on 29 July 2020. 10.1136/bmj.m1091 PMC7190011 32217556

[24] European Society of Cardiology. Position Statement of the European Society of Cardiology (ESC) Council on Hypertension on ACE Inhibitors and Angiotensin Receptor Blockers. https://www.escardio.org/Councils/Council-on-Hypertension-(CHT)/News/position-statement-of-the-esc-councilon-hypertension-on-ace-inhibitors-and-ang. Accessed 7 Aug 2020.

