# Supplementary material for: Morbidity and mortality outcomes of COVID-19 patients with and without hypertension in Lagos, Nigeria: a retrospective cohort study
Source: Glob Health Res Policy. 2021 Jul 29;6:26. doi: 10.1186/s41256-021-00210-6 (PMC8319704; doi:10.1186/s41256-021-00210-6)
Supplement: Supplementary file 1 — Additional file 1: Table S1. Cox regression for risk of death among patients with hypertension compared with patients without hypertension. (adjusting for Age, sex, diabetes mellitus, renal diseases, HIV/HBV co-infection, asthma, other cardiovascular diseases and cancer). Table S2. Distribution of comorbidities in patients with and without hypertension. [file 41256_2021_210_MOESM1_ESM.docx]

**Supplementary Material**

**Title of manuscript: Morbidity and mortality outcomes of COVID-19 patients with and without hypertension in Lagos, Nigeria: A retrospective cohort study**

**Table 1: Cox regression for risk of death among patients with hypertension compared with patients without hypertension.** (adjusting for Age, sex, diabetes mellitus, renal diseases, HIV/HBV co-infection, asthma, other cardiovascular diseases and cancer)

|  | **Unadjusted** | |  | **Adjusted** | |  |
| --- | --- | --- | --- | --- | --- | --- |
|  | **HR** | **95% CI** | **p** | **aHR** | **95% CI** | **p** |
| Hypertension | 6.51 | 4.1 – 10.3 | 0.001 | 1.99 | 1.2 - 3.4 | 0.013 |
| Sex (male/female) | 1.71 | 0.9 – 2.9 | 0.051 | 1.59 | 0.9 - 2.8 | 0.099 |
| Diabetus Mellitus | 8.47 | 5.2 – 13.7 | 0.001 | 2.93 | 1.7 – 5.0 | 0.001 |
| Asthma | 2.06 | 0.6 – 6.5 | 0.218 | 1.75 | 0.5 - 5.7 | 0.354 |
| Cancer | 9.30 | 2.9 – 29.6 | 0.001 | 8.58 | 2.5 – 29.1 | 0.001 |
| Renal Disease | 15.19 | 5.5 – 41.7 | 0.001 | 3.65 | 1.2 - 10.8 | 0.020 |
| Other CVS diseases | 5.57 | 1.3 – 22.7 | 0.017 | 0.77 | 0.2 - 3.5 | 0.736 |
| HIV-HBV | 6.62 | 2.1 – 21.1 |  | 7.08 | 2.2 – 23.1 | 0.001 |
| Age Group(years) |  |  |  |  |  |  |
| < 40 ^R^ |  |  |  | 1 |  |  |
| 40-49 | 1.77 | 0.7 – 4.3 | 0.216 | 1.18 | 0.5 - 3.0 | 0.730 |
| 50-59 | 6.91 | 3.2 – 14.9 | 0.001 | 3.66 | 1.6 - 8.4 | 0.002 |
| >-60 | 21.21 | 10.5 – 42.9 | 0.001 | 8.67 | 3.8 - 19.5 | 0.001 |

R= reference group, HR = Hazard ratio aHR=Adjusted hazard ratio

**Table 2: Distribution of comorbidities in patients with and without hypertension**

| **Variable** | **Hypertensive** | | **Total** | **X^2^** | **p-value** |
| --- | --- | --- | --- | --- | --- |
|  | **Yes** | **No** |  |  |  |
| **Diabetes Mellitus** |  |  |  | 228.79 | 0.001 |
| Yes | 95(25.7) | 55(3.2) | 150(7.2) |  |  |
| No | 274(74.3) | 1647(96.8) | 1921(92.8) |  |  |
| **Total** | **369(100.0)** | **1702(100.0)** | **2071(100.0)** |  |  |
| **Asthma** |  |  |  | 1.051 | 0.305 |
| Yes | 10(2.7) | 32(1.9) | 42(2.0) |  |  |
| No | 359(97.3) | 1670(98.1) | 2029(98.0) |  |  |
| **Total** | **369(100.0)** | **1702(100.0)** | **2071(100.0)** |  |  |
| **Cancer** |  |  |  | 5.077 | 0.036 |
| Yes | 6(1.6) | 9(0.5) | 15(0.7) |  |  |
| No | 363(98.4) | 1693(99.5) | 2056(99.3) |  |  |
| **Total** | **369(100.0)** | **1702(100.0)** | **2071(100.0)** |  |  |
| **Renal disease** |  |  |  | 12.211 | 0.003 |
| Yes | 6(1.6) | 4(0.2) | 10(0.5) |  |  |
| No | 363(98.4) | 1698(99.8) | 2061(99.5) |  |  |
| **Total** | **369(100.0)** | **1702(100.0)** | **2071(100.0)** |  |  |
| **Other Cardiovascular diseases** | |  |  | 35.530 | 0.001 |
| Yes | 11(3.0) | 3(0.2) | 14(0.7) |  |  |
| No | 358(97.0) | 1699(99.8) | 2057(99.3) |  |  |
| **HIV_HBV Co infection** | |  |  | 1.283 | 0.494 |
| Yes | 1(0.3) | 14(0.8) | 15(0.7) |  |  |
| No | 368(99.7) | 1688(99.2) | 2056(99.3) |  |  |
| **Total** | **369(100.0)** | **1702(100.0)** | **2071(100.0)** |  |  |
